# Electrochemical Biosensors for Pathogen Detection: An Updated Review

**DOI:** 10.3390/bios12110927

**Published:** 2022-10-26

**Authors:** Morteza Banakar, Masoud Hamidi, Zohaib Khurshid, Muhammad Sohail Zafar, Janak Sapkota, Reza Azizian, Dinesh Rokaya

**Affiliations:** 1 Dental Research Center, Dentistry Research Institute, Tehran University of Medical Sciences, Tehran 14176-14411, Iran; 2 Health Policy Research Center, Institute of Health, Shiraz University of Medical Sciences, Shiraz 71348-45794, Iran; 3 Department of Medical Biotechnology, Faculty of Paramedicine, Guilan University of Medical Sciences, Rasht 41887-94755, Iran; 4 Department of Prosthodontics and Implantology, College of Dentistry, King Faisal University, Al-Hofuf, Al Ahsa 31982, Saudi Arabia; 5 Center of Excellence for Regenerative Dentistry, Department of Anatomy, Faculty of Dentistry, Chulalongkorn University, Bangkok 10330, Thailand; 6 Department of Restorative Dentistry, College of Dentistry, Taibah University, Al Madinah, Al Munawwarah 41311, Saudi Arabia; 7 Department of Dental Materials, Islamic International Dental College, Riphah International University, Islamabad 44000, Pakistan; 8 Research Center of Applied Sciences and Technology, Kritipur 44600, Nepal; 9 Pediatric Infectious Diseases Research Center (PIDRC), Tehran University of Medical Sciences, Tehran 14197-33151, Iran; 10 Biomedical Innovation & Start-Up Association (Biomino), Tehran University of Medical Sciences, Tehran 14166-34793, Iran; 11 Department of Clinical Dentistry, Walailak University International College of Dentistry, Walailak University, Bangkok 10400, Thailand

**Keywords:** electrochemical, biosensors, pathogen quantification, medical diagnostics, pathogen detection

## Abstract

Electrochemical biosensors are a family of biosensors that use an electrochemical transducer to perform their functions. In recent decades, many electrochemical biosensors have been created for pathogen detection. These biosensors for detecting infections have been comprehensively studied in terms of transduction elements, biorecognition components, and electrochemical methods. This review discusses the biorecognition components that may be used to identify pathogens. These include antibodies and aptamers. The integration of transducers and electrode changes in biosensor design is a major discussion topic. Pathogen detection methods can be categorized by sample preparation and secondary binding processes. Diagnostics in medicine, environmental monitoring, and biothreat detection can benefit from electrochemical biosensors to ensure food and water safety. Disposable and reusable biosensors for process monitoring, as well as multiplexed and conformal pathogen detection, are all included in this review. It is now possible to identify a wide range of diseases using biosensors that may be applied to food, bodily fluids, and even objects’ surfaces. The sensitivity of optical techniques may be superior to electrochemical approaches, but optical methods are prohibitively expensive and challenging for most end users to utilize. On the other hand, electrochemical approaches are simpler to use, but their efficacy in identifying infections is still far from satisfactory.

## 1. Introduction

**Figure 1:**
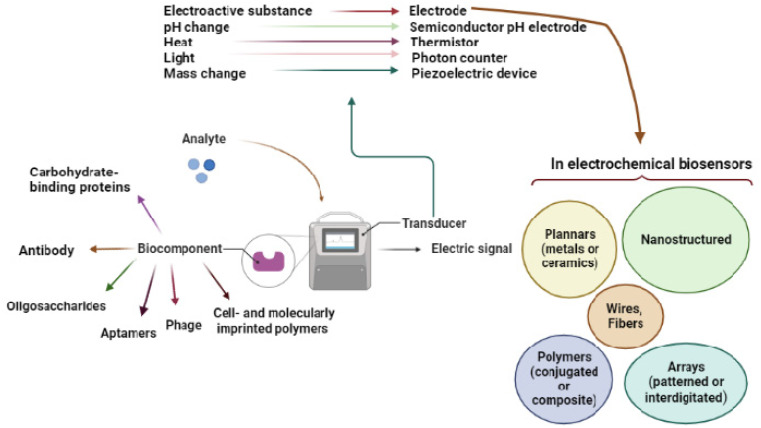
Overview of the biosensor and its components.

Pathogens facilitate the transmission of disease. Fungi, protozoans, and bacteria are only a few of the microorganisms that fall under this category. Pathogens that enter the body via food, drink, and the air affects over 15 million fatalities worldwide [1,2,3]. Virulence and infectious dosage statistics for the COVID-19 virus, a worldwide pandemic, are only beginning to emerge. Rapid and sensitive pathogen detection methods are vital for the treatment of infectious illnesses, and the prevention of illness [4,5,6,7]. Both fluids and aerosols, and surfaces, are covered in this review (Figure 1).

Immunoassays and deoxyribonucleic acid (DNA)-based assays are often used to identify and quantify infections [8,9]. For example, toxin- and species-specific gene sequence data can influence the use of immunoassay or a DNA test at different stages of infection. Immunoassays are regularly used in medical diagnosis and food safety [10]. Immunoglobulins (Igs) are created during and after infection, making them useful for pathogen identification (when the pathogen is gone). These tests involve both the biorecognition component and the target antibody. Immunoassays can be used to detect infections in the body if antigens are made available. Immunoassays can identify infections via antibodies and pathogen epitopes, making them extremely flexible [8,10,11]. Because of the lack of antibodies and the need for extremely sensitive findings, or because the pathogen is present but does not create a significant number of antibodies, DNA-based tests are widely utilized in diagnostics [8,10]. Detecting pathogens that have recently been present in a sample is essential for DNA-based testing to work. Toxins, antibodies, and genes that create toxins can be used to identify pathogens. Toxins, nucleic acids, and viruses are examples of pathogen detection targets. There are many biorecognition components to choose from, from antibodies to aptamers to imprinted polymers [12,13,14]. Enzyme-linked immunosorbent assay (ELISA) [15] and polymerase chain reaction (PCR) [16,17] have been extensively studied for the detection of infections. 

Because of its high sensitivity and specificity, applicability in monitoring, early detection of biothreat agents, and antimicrobial resistance profiling, PCR technology (conventional and real-time PCR) is most frequently utilized in pathogen detection [18,19]. However, it has shortcomings, including the inability to distinguish between infections with identical genetic composition. For instance, when PCR has been used to identify *Listeria monocytogenes* [20] and *Bacillus cereus* [21], respectively, false signals of *Listeria innocua* and *Bacillus thuringiensis* have been recorded [22]. The inability of PCR to distinguish between the DNA of dead and living cells is another significant drawback, and this issue is crucial for the food sector, regulatory bodies, and the customer [19,22]. 

ELISA demonstrates the following benefits: (1) a straightforward process using affordable equipment; (2) high sensitivity and specificity because of an antigen-antibody response; (3) high efficiency since many analyses can be run simultaneously without extensive sample pre-treatment; (4) generally safe and environmentally benign because no radioactive materials or significant quantities of organic solvents are needed; and (5) as low-cost reagents are utilized, the assay is cost-effective. ELISA, however, has the following drawbacks: (1) antibody preparation is time-consuming and costly because it requires a sophisticated technique and expensive culture cell media to produce a particular antibody; (2) a high likelihood of erroneous positive or negative results exists because the surface of the microtiter plate immobilized with antigen has not been sufficiently blocked; (3) antibody instability exists because an antibody is a protein that needs to be transported and stored in a refrigerator; and (4) it has restricted use in foods with a solid matrix or that are viscous, such as peanut butter, jam, and honey [22,23].

Even though label-free biosensors for pathogen detection can be useful for monitoring, they have seldom been reviewed. An analytical system is used in conjunction with a specific biorecognition element, such as a molecular probe, to measure one or more components of a sample. Although they can be extremely sensitive and robust, these testing methods are destructive. They need significant sample preparation and the addition of reagents, which prolongs the time it takes to obtain findings. The presence of background species in a sample can also inhibit bioanalytical techniques, such as PCR [24,25,26], increasing the amount of error introduced into the measurement process [27,28]. Plate-based bioanalytical systems have limitations and need continual real-time monitoring across several applications; hence, other bioanalytical processes should be investigated. 

Merging of targeted biorecognition elements with very sensitive transducer components improves pathogen detection and quantification in biosensors. The International Union of Pure and Applied Chemistry (IUPAC) has said that, to create an effective biosensor, an element that may be directly connected to the biorecognition element must be included [29]. Although the biosensor can measure everything from droplet sizes to continuous flow forms, it must also be a self-contained, integrated instrument. Biosensors that can detect pathogens in real-time without sample preparation may now be used in several settings. A wide range of matrices and conditions may now be analyzed using biosensors, including food and body fluids, as well as surfaces of objects [30]. Biosensors allow both sample preparation-free and label-free approaches [31,32,33,34]. Examples of molecular species known as “reporters” include organic dyes and quantum dots. Biorecognition elements or secondary binding stages can be used to directly affix labels to a target or through a succession of sample preparation operations or secondary binding stages [35]. Consequently, label-free biosensors do not rely on a reporting species to detect the target species [36,37]. Using a label-free assay means fewer sample preparation steps and lower costs than using a label-based assay, both of which are key factors in applications with limited preparation facilities or trained personnel [36,37,38].

Pathogen biosensing transducers of many sorts have been examined [13,26,38]. Either mechanical or optical transducers, such as cantilever biosensors or surface plasmon resonance (SPR)-based sensors can be used to detect infections [39,40,41]. A transducer consisting of conducting or semiconducting materials is used. An electrochemical approach can be used to convert the chemical energy released by pathogens and electrode-immobilized biorecognition components into electricity. Biosensors based on electrochemical processes are able to detect pathogens without the need for sample preparation, enabling in situ detection of pathogens on surfaces, quick and low-cost pathogen detection platforms, multiplexing pathogen detection, and wireless data collection actuation gathering (Table 1) [39,42,43,44,45,46]. Table 2 shows the chronological order of using biosensors to detect bacteria or viruses.

Application of real (sometimes complex) samples at the point-of-care (POC) and in the field is one of the issues that researchers are still attempting to solve for all types of biosensors. The type of instrument required to advance electrochemical biosensors to point-of-care has been made available by screen-printing technology [81]. Because of their reliability, reproducibility, mass production, and low cost, screen-printed electrodes (SPEs), which first debuted in the 1990s, have significantly contributed to the advancement of electrochemical biosensors [81,82,83]. SPEs have been found to be flexible tools that could be molded into many shapes, manufactured of various materials, and modified with a range of biological components, including enzymes, antibodies, DNA, synthetic recognition elements, and others [82]. Additionally, when using the enhanced electrocatalytic characteristics of nanoparticles, modifications with a variety of nanomaterials and synthetic recognition elements have been used to increase sensitivity [82]. For instance, nanomaterials (carbon nanotubes, graphene, gold nanoparticles, etc.) applied on an SPE’s working electrode (WE) can greatly increase surface activity due to their superior electrocatalytic capabilities and substantially larger specific surface area. This technique can be carried out automatically on the planar SPE by a mass-producible dispenser or just before mixing the modifier with the ink while printing. One benefit of this improvement is that it can aid in the direct detection of some conductive analytes. In addition, it is frequently used to enhance the immobilization of the recognition element, which is frequently a biomolecule, to facilitate analyte identification and signal transduction [84].

Additionally, the appealing characteristics of carbon—chemical inertness, low background currents, and a broad potential window—have attracted a large amount of attention to SPEs. In addition to carbon, which is still the most affordable option, other metals such as gold also have advantages. The suitability of gold SPEs in electrochemical biosensors has been greatly increased by the affinity between thiol moieties and gold, which enables SPEs with gold working electrodes to be easily adjusted with the production of self-assembled monolayers [81].

This review analyzes all electrochemical biosensor pathogen detection aspects, including the design, manufacturing, measurement format, and performance. Research on electrochemical biosensors for pathogen detection will address current technological and methodological issues and new application fields. 

## 2. Pathogen Detection with Electrochemical Biosensors

One of the most common functions of chemical sensors is to produce an analytically useful signal [29]. Electrochemical biosensors uniquely detect their targets. Biosensor-based pathogen detection strategies and the electrochemical approach have several other characteristics in terms of sample processing and sensor-specific actions. The following section discusses transduction, bio-recognition, and measurement formats for electrochemical biosensors.

Generally, pathogens can be identified through the presence of generated antibodies in an organism, which may be present both during and after an infection. In such assays, both the biorecognition element and the target are antibodies. Electrochemical biosensors combine an analyte-receiving mechanism and an electrochemical transducer, where the interaction between the targeted analyte and the transducer generates an electrochemical signal in current, potential, resistance, or impedance format [85,86]. There is a wide range of electrochemical biosensor schemes with different signal mechanisms, e.g., differential pulse voltammetry (DPV), voltammetric cyclic voltammetry (CV), polarography, square wave voltammetry (SWV), stripping voltammetry, alternating current voltammetry (ACV), and linear sweep voltammetry (LSV). Furthermore, electrochemical biosensors can use different types and forms of nanomaterials, nanoparticles, and nanocomposites to enhance the sensitivity of the detection mechanisms and to provide better detection limits through different strategies [87,88].

### 2.1. Transduction Elements

**Figure 2:**
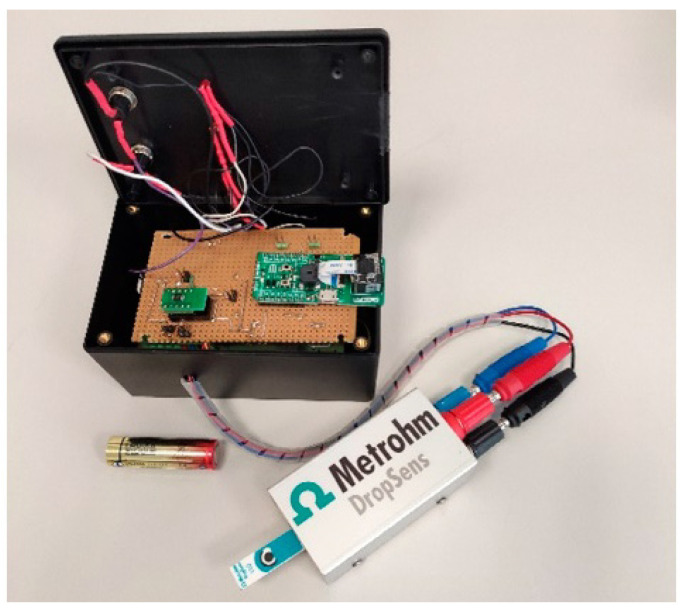
The smart portable wireless potentiostat for Rapid Quantification of SARS-CoV-2 Spike Protein. Reprinted with permission from ref. [80].

The working electrode is often the principal transduction element when employing an electrochemical biosensor. Conventionally, a three-electrode potentiostat system employs three electrodes; however, conductometry and impedance measurements often utilize two electrodes (working and auxiliary). Manufacture of electrodes can use a range of materials and procedures. Electrons and holes pass through an electrode to transfer charge. Electrodes are made from conductive and semiconducting materials such as gold (Au) and carbon (C). Different manufacturing methods may be utilized to make electrodes of different sizes. For instance, Fortunati et al. recently quantified the SARS-CoV-2 spike protein using an Internet of Things-Wifi (IoT-WiFi) smart and portable electrochemical immunosensor (Figure 2) with integrated machine learning characteristics. Based on the immobilization of monoclonal antibodies against the SARS-CoV-2 S1 subunit on screen-printed electrodes (SPEs) functionalized with gold nanoparticles, the immunoenzymatic sensor was developed. The working electrode diameter of the SPEs was 4 mm, and their dimensions were 3.4 × 1.0 × 0.05 cm. The counter electrode was made of carbon, while the reference electrode and electrical connections were silver [80].

An electrode’s material type, manufacturing method, and design all play a role in categorizing it. The form factor may be used to classify electrode designs into planar, wire, nanostructured, and array-based types of structures. A biosensor’s ability to detect a specific biological agent, and its sensitivity and dynamic range, are determined by the electrode’s shape and characteristics and the material, production technique, and design of the biosensor. As a result, the entire cost of the biosensor is significantly affected [89].

#### 2.1.1. Metal Electrodes

The detection of pathogens has traditionally relied on gold (Au) and platinum (Pt) electrodes. A cutting process is commonly used to create thick metal electrodes. Traditional microfabrication methods, such as physical vapor deposition and screen printing, are frequently used to make thin-film metal electrodes [90,91]. Transducer elements are often built using Teflon, polyether ketone (PEK), and glass as insulating polymers or ceramic substrates for the resulting conductive components. Pathogen detection applications have yet to utilize 3D printing technologies such as inkjet printing [92,93,94]. Selective laser melting and microextrusion printing have also been employed to manufacture electrochemical sensors and electrodes. The detection limits of unstructured metal electrodes can vary widely. These biosensors for bacteria have detection limits of 1 to 10^4^ CFU/mL, for example, using unstructured metal electrodes [95,96,97].

#### 2.1.2. Ceramic Electrodes

Pathogens in food may be detected using semiconducting and conducting ceramics such as indium tin oxide (ITO), polysilicon, and titanium dioxide (TiO_2_). A silicon electrode was used by Das et al. to detect *Salmonella typhimurium* (*S. typhimurium*) [61]. Antibody-functionalized indium tin oxide (ITO) electrodes developed by Barreiros dos Santos et al. can detect *E. coli*, according to researchers [98]. Specifically, ITO’s high conductivity and transparency directly correlate with biosensor response and pathogen surface coverage [99,100]. Due to ITO’s high conductivity and transparency, biosensor response and pathogen surface coverage are linked [101].

#### 2.1.3. Polymer Electrodes

Pathogen-detecting electrodes have also been made from polymers. Polymers are not just good for human health and the environment, but are also relatively low-cost. Various biorecognition element immobilization techniques are also compatible with polymer electrodes [102,103]. Implantable and wearable biosensors require electrode–tissue mechanical matching, which is made possible by polymers’ mechanical properties. An (organic) conjugated polymer (CP) electrode or a polymer composite can be categorized as a type of polymer electrode, and they have long been used for pathogen detection [104,105,106]. 

Remarkable transparency, biocompatibility, low oxidation potential, outstanding conductivity, ease of fabrication, low cost, and a small band gap (e.g., 1.6 eV) are some of the special qualities of organic CPs [107,108,109]. Poly (acetylene), poly (pyrrole), poly(thiophene), poly(terthiophene), poly(aniline), poly(fluorine), poly (3-alkylthiophene), poly tetrathiafulvalene, poly naphthalene, and poly (p-phenylene sulfide), poly(para-phenylene vinylene) are examples of common types of organic CPs [107,109].

*E. coli* and human influenza A virus were detected using spin-coated films coated with poly (3,4-ethylene dioxythiophene) [76]. Organic CPs’ semiconducting nature gives them unique optical and optoelectronic capabilities. Thus, synthetic chemists’ capacity to modify the chemical structures of polymerized monomers allows for the design and tweaking of CPs for particular purposes [109].

Polymer composite electrodes, which comprise a nonconductive polymer combined with a conductive one, frequently conduct or convey scattered words. Dispersed phases such as graphite or gold nanoparticle (AuNP)-graphene or carbon nanotubes (CNTs) have been widely employed in conjunction with different polymers, including poly ethyleneimine (PEI), poly allylamine (PA), and chitosan (PAA) [110,111,112,113]. 

Researchers have developed a poly allylamine/CNT polymer composite electrode that may be used for anodic stripping voltammetry to detect bacteria including *E. coli*, *S. typehimurium,* and *Campylobacter* at concentrations as low as 10^3^–10^5^ cells/mL [113]. *S. typhimurium* was detected using AuNP-coated synthetic polymer composite electrodes made of poly (amidoamine), carbon nanotubes, and chitosan [110,114]. 

Polymer composite electrodes have a detection limit of 1–10^3^ CFU/mL, which is equivalent to that of metal and polymer electrodes. Nanomaterials may be disseminated throughout the polymer in polymer composite electrodes rather than using electrode nanostructuring methods. Polymer electrodes have risen in popularity because of the growing need for flexible biosensors. Electrodes consisting of a polymer or a film that may be affixed to a flexible substrate, such as paper, are among the most recent biosensor technologies being researched [115], because 3D printing processes are compatible with conjugated polymers and composites of polymers [116,117]. Additionally, polymer electrodes are becoming attractive candidates for wearable biosensors that can conform to the wearer’s body [118,119]. Regarding polymer electrodes, the most common form factor is a thin film, but nanowires and nanofibers can also be used [119,120]. 

#### 2.1.4. The shape and Design of the Electrodes

Electrodes made from Au have been utilized to detect infections of all forms and sizes. Advanced masks and programmable tool paths can be utilized to create electrodes using lithography and 3D printing [121,122]. In addition to complicated form factors, electrode patterning may be used to fabricate electrode arrays using lithographic, 3D printing, and assembly techniques [122]. Biosensor sensitivity and multiplexing have been improved by electrode arrays, which include interdigitated microelectrodes and other patterned electrodes. There are alternating, parallel fingers on the electrodes in an interdigitated array microelectrode (IDAM) with excellent response time [123]. For pathogen detection, Au interdigitated microelectrode arrays are a popular choice. 

*S. typhimurium* can be detected using electrochemical impedance spectroscopy (EIS) using interdigitated Au micro electron arrays, such as those used by Dastider et al. [70]. Detection of *S. typhimurium* has also been performed utilizing interdigitated arrays of ceramic electrodes such as ITO [66,124]. Electrode arrays with geometries other than interdigitated designs can be made using the aforementioned emerging manufacturing processes for electrochemical sensing applications. Arrays of silver (Ag) microelectrodes may be created using aerosol jet additive manufacturing [125].

#### 2.1.5. Electrode Nanostructuring

Nanotechnology represents a multidisciplinary field that covers materials and devices’ design, fabrication, and functionality with dimensions in the nanometer (nm) domain [126]. Sensitive biosensors can be made using transducers whose physical dimensions are similar to those of the target species [127,128]. A wide variety of electrode sizes, ranging from micro- to nanometer, have been studied in the search and nanoscale planar electrodes are among the most common methods [10,129]. Nanomanufacturing techniques, such as making nanowires from the bottom up and from the top down, have produced pathogen detection electrodes with nanostructures [130]. 

Nanomanufacturing processes from the bottom up and from the top down have both been used to make nanowire-based electrodes [131]. Nanowires can even have triangular cross-sections. When addressing the length-to-width ratio, nanowire aspect ratios from 1 to more than 10 are possible [132,133].

**Figure 3:**
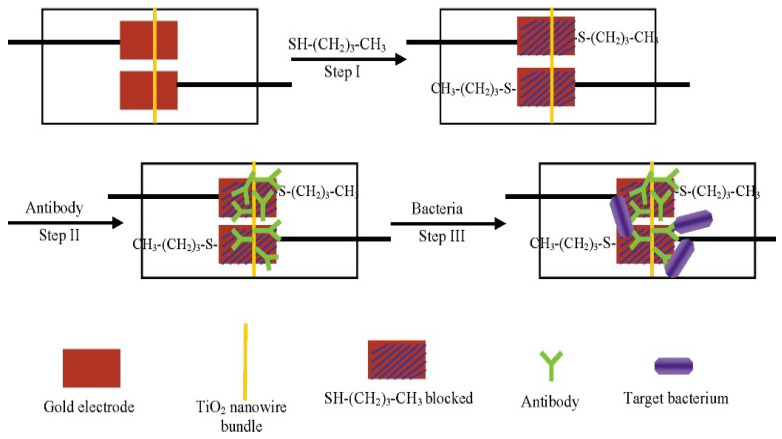
Principle of detection of bacteria such as *L. monocytogenes* using a TiO2 nanowire bundle microelectrode-based impedance immunosensor. Reprinted with permission from ref. [60]. Copyright 2008, American Chemical Society.

**Figure 4:**
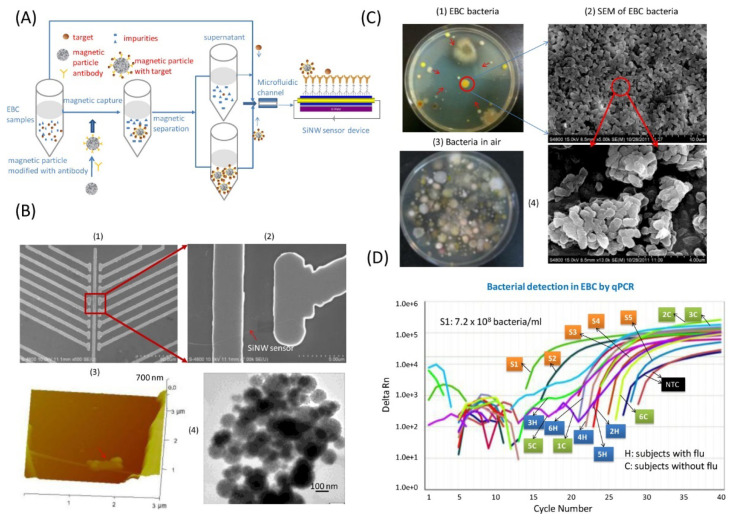
(**A**) Shows an experimental method for detecting Influenza virus and 8 iso Prostaglandin F2α (PGF 2a) in exhaled breath condensate (EBC) samples using a silicon nanowire (SiNW) sensor device with and without magnetic concentration. The EBC samples were collected, diluted 100 times, and then flowed at a rate of 170 L/min to the sensor device. (**B**) SiNW sensor apparatus used in the sensing tests: (1) an optical image of the chip device; (2) a scanning electron microscopy (SEM) image of a single SiNW sensor; (3) an atomic force microscopy (AFM) image of a SiNW device modified with an anti-H3N2 virus antibody and infected with viruses; and (4) SEM photos of magnetic beads. (**C**) Bacteria found in indoor air and EBC samples taken from human participants with and without the flu (Influenza virus). Following culture, bacteria were found in the following samples: (1) EBC samples; (2) SEM images of the bacteria present in the samples; (3) indoor air samples; and (4) higher resolution SEM photographs of the bacteria present in the samples (2). (**D**) The use of quantitative PCR (qPCR) to identify bacteria in EBC samples obtained from flu patients and healthy individuals. Reproduced with permission from ref. [134]. Copyright 2012, American Chemical Society.

Detection of pathogens by use of metallic and ceramic micro and nanowire electrodes has been investigated. For instance, TiO_2_ electrodes for the detection of *Listeria* (*L. monocytogenes*) were created by Wang and colleagues using a bottom-up wet chemical technique (Figure 3) [60]. Moreover, an array-based file format of the human influenza virus was detected using a chemical vapor deposition approach developed by Shen and colleagues (Figure 4) [134].

In another study, label-free chemiresistive sensors based on a nanoribbon made of polypyrrole (PPy) through a lithographically patterned nanowire electrodeposition (LPNE) technique were used to detect cucumber mosaic virus [135]. 

**Figure 5:**
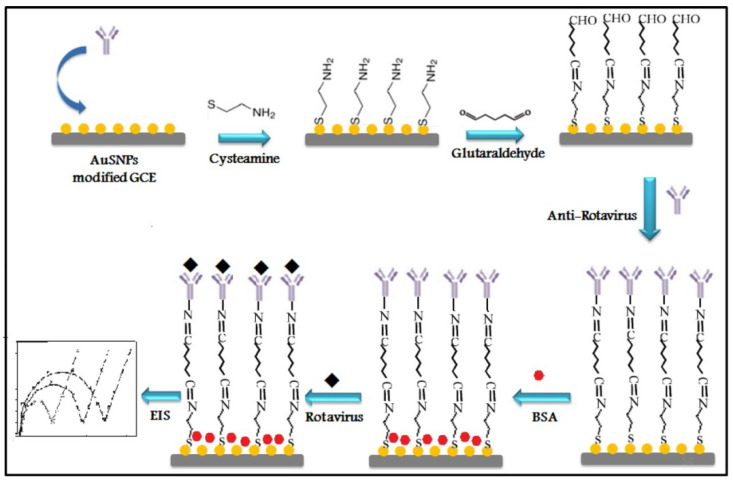
Schematic illustration of the stepwise preparation of anti-rotavirus self-assembled monolayers (SAM) immunosensor (BSA: Bovine Serum Albumin). Reprinted with permission from ref. [74]. Copyright 2016, John Wiley & Sons, Inc.

Electrode surfaces have also been studied for infection detection using micro and nanostructured characteristics. Nanostructuring allows for an increase in electrode surface area without a corresponding rise in electrode volume [136]. The electrical characteristics of electrodes can be affected by changes in the electrode’s surface topography. Reduced electrical resistance across a wide frequency range benefits neural monitoring and recording applications when using poly (3, 4-ethylenedioxythiophene) (PEDOT) on silicon electrodes [137]. Pathogen-detecting nanostructures that go beyond the bottom-up wet chemistry and electrochemical techniques used to create nanowire-based electrodes are being created. Electrode nanostructures can be created via wet chemical processes [138]. In many cases, nanoparticles are deposited or coupled to planar electrodes to create nanostructured electrodes. Nanostructured surfaces for biorecognition elements can be created by depositing AuNPs on planar electrodes. In these studies, physical adsorption processes are used to affix the particles to the planar electrode [74] or chemical methods [139]. Electrode nanostructuring can benefit from CNTs and AuNPs, according to a growing body of research. For instance, Attar et al. [74] developed a straightforward and accurate label-free assay for rotavirus detection utilizing electrochemical impedance spectroscopy (EIS). Cysteine monolayers self-assemble on a glassy carbon electrode that has been modified with gold sono nanoparticles (AuSNPs) to create the immunosensor (Figure 5).

The aggregation of biorecognition elements on high-curvature nanostructured gold microelectrodes was shown to be less than on flat electrodes in DNA sensing experiments combining experimental research and molecular dynamics simulations, as revealed by De Luna et al. [140]. Researchers used carbon nanoparticles to identify the Japanese encephalitis virus [47].

Recognition of pathogens has been made possible via electrochemical nanostructuring and bottom-up electrochemical techniques. Gold (III) chloride hydrates were electrochemically deposited onto a nanostructured gold electrode to detect norovirus in lettuce extracts [129]. Nanoporosity, a type of electrode porosity, allows for electrode nanostructuring in addition to the more typical way of depositing materials on planar electrodes. Nguyen and colleagues used platinum (Pt) microwires coated with nanoporous alumina to look for the West Nile virus. [62]. Nanostructured electrodes have been proven to boost biosensor sensitivity and the limit of detection (LOD) [129,141]. The fluctuation in electrode nanostructure quality from device to device and batch to batch still needs to be understood. Nanostructured surface features (such as topography and structure) and material qualities may differ among mass-produced electrodes. Biosensor repeatability may also be affected by this variability in nanostructure quality; however, this is not yet discovered. 

#### 2.1.6. Complimentary Transduction Components

Pathogen detection applications have also looked at biosensors with integrated electrodes and complementing transducers, given the necessity for quick and reliable readings. It is possible to concurrently monitor fluid mixing and molecule binding events with the use of electrodes and transducers [142]. Biosensors having multiple transducers, such as hybrids, allow for in situ target binding verification and complement analytical observations. 

Hybrid electrochemical biosensors have been coupled with optical and mechanical transducers for pathogen detection. In Electrochemical Optical Waveguide Lightmode Spectroscopy (EC-OWLS), optical and electrochemical sensing are combined into a single apparatus [143]. With EC-OWLS, it is possible to monitor electrode surface changes and development [144]. Pathogens may be identified by using this method. Electrochemical sensing is combined with surface plasmon resonance to detect changes in the electrode–electrolyte (conductive interface) interface refractive index caused by binding (surface plasmon resonance (SPR)) [145]. These functions can be tracked with this technology but can also identify infections with better accuracy [146]. Complementary responses can also be used to confirm the presence of binding functions [147]. Using mechanical transducers’ principal radiation effects, pathogens may be identified both at the conductive contact and in the bulk solution [121]. Secondary transducers can be used to provide force to constrain or catch targets. Shear-mode resonators and cantilever biosensors are useful in removing surface-bound proteins in various investigations [148,149]. Hybrid designs might help biofouling-prone electrodes.

Some biosensor-based pathogen detection methods are based on coupling electrochemical biosensors with a standard biological methodology. For example, an electrochemical-colorimetric biosensor is a biosensor that combines electrochemical-colorimetric (EC-C) biosensing methods. Target-to-active species reactions are detectable via electrodes or colorimetric transduction pathways. AuNP-modified ITO electrodes with monoclonal antibodies and dual-labeled magnetic beads were utilized to detect human Enterovirus 71 in the EC-C method by Hou et al. [150]. Using magnetic nanobeads tagged with antibodies and Horseradish Peroxidase (HRP), a second binding step was performed after exposing the electrode to enterovirus-containing samples. Color, fluorescence, and luminescence detection are all common uses for laser-active labels. The most advantageous aspects of employing magnetic fields are that they can be tuned by varying the applied current on the micro-conductors, and that they can be applied externally or from integrated micro-conductors. Furthermore, high-gradient magnetic fields that can be measured by magnetic sensors can be used to modify the magnetic markers inside microfluidic channels [151]. Organic fluorophores that are not protein-based include fluorescein and rhodamine. A wide variety of quantum dots, including CdS, CdSe, and GaAs, are frequently employed in the design of electronic devices [152,153]. Detection of the target species is covered in the next sections.

### 2.2. Biorecognition Elements

This section covers the components of electrochemical biosensor pathogen detection. Biorecognition elements used to identify infections and the methods used to attach them to electrodes in a biosensor are the subject of our next discussion. The biocatalytic properties and biocomplexity of biorecognition components can be used in electrochemical biosensors. Biological biorecognition components employ macromolecule-catalyzed reactions in their biosensor responses. One of the most common biocatalytic biorecognition components is enzymes. Various chemical sensing applications can benefit from enzyme-based biorecognition elements, although pathogen detection is the most common usage. When analytes interact with macromolecules or structured molecular assemblies, biocomplexity biorecognition elements are triggered. To identify infections, the body relies on biocomplexity biorecognition components such as antibodies, peptides, and phages.

Lately, modified molecules including DNAzymes, peptide nucleic acids, and molecules that suffer a selective screening, such as aptamers and peptides, are gaining interest due to their biorecognition capabilities and other advantages over purely natural molecules, such as robustness and lower product costs. Antimicrobials with a broad- spectrum activity against pathogens, similar to antibiotics, are also used in dual diagnostic and remedial strategies. 

Other successful pathogen identification strategies use chemical ligands, molecularly imprinted polymers, and Clustered Regularly Interspaced Short Palindromic Repeats-associated nuclease [154].

#### 2.2.1. Antibodies and Antibody Fragments

Electrochemical biosensors, such as antibodies and antibody fragments, frequently incorporate these pathogen-detection biorecognition components. Immunosensors are biosensors that use biorecognition elements based on antibodies. Due to their high affinity and selectivity for target species, antibodies serve as the gold standard in pathogen detection as the gold standard biorecognition element. An epitope is an antigen region recognized by antibodies as a specific binding site [130]. Using fluorescent or enzymatic tags, antibodies can be labeled as label-based techniques. As a result of the additional reagents and processing steps needed for label-based approaches, there are measurement limitations [33,36]. The biosensor’s selectivity may also be affected by antibody labeling, which alters the binding affinity to the antigen. Pathogen detection methods based on label-based biosensing have previously been discussed in depth elsewhere [155,156].

Selected pathogen detection is made possible by using both monoclonal and polyclonal antibodies [130]. The production process, selectivity, and binding affinity all vary. Hybridoma technology is used to create monoclonal antibodies [157]. Because of their high specificity and affinity for a particular epitope, monoclonal antibodies are more resistant to cross-reactivity. Monoclonal antibodies are better at identifying specific targets. Immunoglobulin proteins can be isolated from contaminated blood and used to produce polyclonal antibodies [157]. A single antigen can be specifically targeted by polyclonal antibodies, which are generated in large quantities and have particular epitopes. Because of the larger batch-to-batch variability of polyclonal antibodies than monoclonal antibodies, they are more expensive to produce but may be used in wider applications [158]. Low-temperature storage is one of the drawbacks of antibody use, as is the high cost. Pathogen detection relies on biorecognition elements. A monoclonal antibody is the most common biosensing element utilized in testing, including secondary binding stages, whereas polyclonal antibodies are used secondarily to assist in target marking. Immobilized biorecognition components, such as polyclonal antibodies, are often utilized in pathogen detection tests that do not require further binding processes.

Single-chain variable fragments (ScFvs), smaller and less bulky versions of antibody fragments, exhibit selectivity that is very close to that of antibodies. Half-antibody fragments in pathogen detection have increased biosensor sensitivity without sacrificing selectivity [159]. Re-engineered IgGs, dimers, ScFvs, and Fragment Antigen-Binding (Fab) regions are potential biorecognition components for pathogen detection [158].

#### 2.2.2. Carbohydrate-Binding Proteins

Lectins and other carbohydrate-binding proteins are useful for pathogen detection because they can bind ligands unique to the target organism. Low-cost, high-yield automated synthesis processes allow the production of peptide biorecognition elements, which can be modified [160]. Many studies have examined the ability to detect *E. coli* using lectins such as Concanavalin A (ConA) [41,77]. Yet, pathogen detection using electrochemical biosensors has not been thoroughly studied. Breast cancer cells may be detected in real-time using oligopeptides, according to Etayash et al. [161].

#### 2.2.3. Oligosaccharides

Infectious disease pathogens have receptors for carbohydrate-specific trisaccharides, a form of carbohydrate that can interact with these receptors. For the detection of illnesses, trisaccharide ligands have been used in electrochemical biosensors [162]. Biosensors coated with hemagglutinin-targeted trisaccharide ligands have been used to detect the influenza A virus in humans (H1N1) [51]. It is difficult to employ carbs as biorecognition components because of the limited specificity and low affinity of carbohydrate–protein interactions [163].

#### 2.2.4. Oligonucleotides

**Figure 6:**
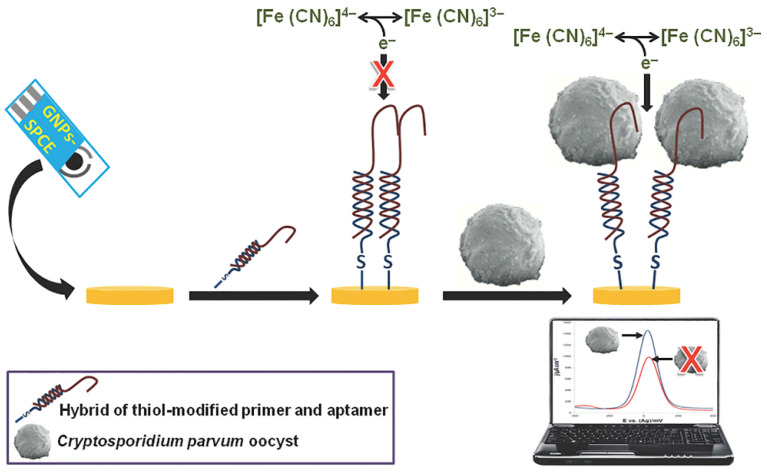
Schematic illustration of the electrochemical detection method used for the detection of *Cryptosporidium parvum* Oocysts. Self-assembling aptamer and primer hybrids were applied to a carbon electrode that had been screen-printed with gold nanoparticles (GNPs-SPCE). By using square wave voltammetry, the binding of the *C. parvum* oocyst to the immobilized aptamer increases the redox current. Reprinted with permission from ref. [168]. Copyright 2015, PLOS.

Single-stranded DNA can be utilized as a biorecognition element to detect diseases (ssDNA). Regarding pathogen detection, ssDNA aptamers are widely utilized in electrochemical biosensors, whereas ssDNA is more commonly seen in DNA-based tests. These single-stranded oligonucleotides have strong binding affinity and selectivity for a large variety of different compounds [164,165]. It is possible to separate aptamers from vast random sequence pools using Systematic Evolution Of Ligands By Exponential Enrichment (SELEX) [166]. Extracting and amplifying binding sequences is feasible using a random oligonucleotide sequence pool. Aptamers can be narrowly targeted to the molecules designed to interact with [166]. An additional benefit of aptamers is their lower production cost compared to antibodies and other biorecognition components [167]. Ten rounds of SELEX were utilized for fruit samples by Iqbal and colleagues to discover 14 high-affinity aptamer clones for *Cryptosporidium parvum* (Figure 6) [168].

As mentioned, the main alternatives to antibodies being studied are derivatives of nucleic acids such as aptamers, PNAs, DNAzymes, and antibody-derived fragments. The chief benefit of those molecules is that they can be established much more cheaply than antibodies. For example, they have proven target affinity comparable with their antibody counterparts while exhibiting excellent stability and reproducibility, which is a vital requirement for POC diagnostics. They are also adaptable enough to be combined with most POC sensor detection platforms, including electrochemical, optical, colorimetric, and Lateral Flow Assay (LFA). Their main drawback is that they require a selection process that is time-consuming and sometimes problematic to perform.

Regarding their wide range, they are usually used for pre-enrichment or combined with other biorecognition elements to improve the sensitivity of the sensor. A huge number of AMPs are under scrutiny; however, their emerging disadvantage is the lack of selectivity [155].

The degradation of aptamers and the possibility of cross-reactivity and repeatability when using different processing processes have not yet allowed them to totally replace traditional bio-recognition components like antibodies [103].

#### 2.2.5. Phages

Bacteriophages (bacteriophages) are viruses that infect and proliferate within bacteria by the usage of tail-spike proteins, which connect to particular receptors on the host cell [169,170]. Pathogens can be detected using electrochemical biosensors based on these identification components [155,171,172,173]. Bacteriophages’ appearance, selectivity, and structure are categorized into several classes. These biosensors use bacteriophage electrochemical biosensors to detect pathogens. For example, Shabani and colleagues studied selective impedimetric detection with *E. coli*-specific T4 bacteriophages [174]. In the study of *E. coli* detection, Mejri et al. examined bacterial phages and antibodies as biorecognition components. The biosensor’s water stability and sensitivity were enhanced by a factor of four when bacteriophages were used instead of antibodies in that investigation, as evidenced by the EIS measurements [175]. As much as 10^5^ CFU/mL of *L. innocua* could be detected using screen-printed gold electrodes that were coated with peptidoglycan hydrolase immobilized bacteria. Researchers have demonstrated that bacteriophages can be effective biorecognition components in water monitoring that need continual liquid testing [67].

#### 2.2.6. Cell and Molecularly Imprinted Polymers

Developed ScFvs are synthetic molecular biomarkers. Contrary to popular belief, biorecognition components based on materials use morphology particular to the target organism to gather data. Molecularly imprinted polymers (MIPs) and surface imprinted polymers (SIPs) are biorecognition strategies most often used in biomaterials research. Bacterial lithography, micro-contact stamping, and colloid imprinting have all been used to make cell-imprinted polymers (CIPs) and MIPs [176,177]. As indicated in the study of Jafari et al., an impedimetric approach was utilized to detect *E. coli* utilizing sol-gel films of tetraethoxysilane (TEOS) and (3-mercaptopropyl)trimethoxysilane (MPTS) [75,178]. Biosensors may be regenerated using MIPs and CIPs. MIPs and CIPs, molecular biorecognition elements, should be employed instead of structurally altering components to prevent regeneration. CIPs and MIPs are frequently regarded as less selective for the target than antibodies because of their poor chemical selectivity [179,180].

#### 2.2.7. Clustered Regularly Interspaced Short Palindromic Repeats/Associated Nuclease (CRISPR-Cas)

The main advantage of CRISPR is that its single-base resolution selectivity is unmatched by any other biorecognition element. The nature of the technology enables it to be leveraged in POC diagnostic sensing platforms.

SARS-CoV-2 can be detected in saliva droplets and nasal discharge. Saliva is a reliable tool to detect SARS-CoV-2 by RT-rPCR analysis [181]. For the detection of SARS-CoV-2, Hou et al. [182] proposed an alternative to the standard reverse transcription-quantitative polymerase chain reaction (RT-qPCR) detection using a rapid assay based on polymerase-mediated amplification and CRISPR/Cas13a. This isothermal method is highly advantageous because it does not require expensive and bulky thermocycler equipment and only takes 40 min. Subsequently, CRISPR gRNAs and RPA primers were designed and screened. A primer set that targeted open reading frame 1ab (orf1ab) displayed the best specificity and sensitivity and was used to develop the CRISPR assay based on T7 transcription and a Cas13 detection step.

To evaluate the specificity of the CRISPR assay, target viral DNA was substituted with human DNA and a panel of bacterial and viral pathogens. None of these test samples caused a false positive reaction. Further, the CRISPR assay demonstrated 100% sensitivity. In the future, the role of CRISPR-associated nucleases can be expanded for direct diagnostic testing of nucleic acids due to their exceptional single-molecule sensitivity [154].

#### 2.2.8. Antimicrobial Peptides

Antimicrobial peptides (AMPs), as biorecognition elements, belong to the innate immune system of living organisms and are very effective in interacting with bacterial membranes. They offer unique advantages compared to other classical bioreceptor molecules such as enzymes or antibodies. Moreover, impedance-based sensors allow the development of label-free, rapid, sensitive, specific, and cost-effective sensing platforms. AMPs and impedimetric transducers combine excellent properties to produce robust biosensors for the early detection of bacterial infections.

### 2.3. Biosensing and Surface Immobility

Surface modification of the electrode is crucial to biosensor performance due to the functional mechanism of biosensors. Biosensing components must be mounted on electrodes. An irreversible bond is formed between the target species, the biorecognition element, and the electrode when they are both impermeable. Electrochemical pathogen detectors generally employ proven methods to prepare the biorecognition layer.

To immobilize enzymes on an electrode using a polymer layer is the simplest and most used method [78]. However, there are two fundamental drawbacks to this approach. One is that the enzymes’ activity can be altered due to structural changes brought on by the polymer layer, which the layer’s pH can modify. The other issue is that the biosensor’s reaction time and sensitivity can be compromised since the thickness of the polymer layer cannot be accurately regulated. Some research groups have successfully used neutral pH polymers, including silicate sol-gel, for enzyme immobilization to avoid these drawbacks and maintain enzyme function [183]. In addition, surface modification frequently involves nanomaterials such as carbon nanotubes (CNTs) and metal nanoparticles to achieve higher performance. The difficulties in assessing and comparing the various modification procedures and determining the ideal one have emerged because of the vastly varying biosensor performance that results from each. To address this issue, a standardized procedure is needed that compares the efficacy of biosensors made using various techniques [184]. Immobilization and surface passivation technologies are thoroughly investigated in this review while explaining methods.

In recent years, electrochemical biosensors have been used extensively as an alternative to conventional methods for the detection of pathogens because of their high sensitivity, fast response time, and low cost. They exhibit more versatile detection systems, which provides various applications that are capable of real time quantification. Despite the advantages of electrochemical biosensors, there are some issues regarding the analyte detection. One of the issues to be considered for pathogen detection is that the developed biosensor system should allow multiple detection. In addition, detection of pathogens is a significant challenge in medicine due to their vast number and variety [85]. The consistency of the fabricated biosensors is greatly affected by the surface condition of the electrodes and the unspecific absorption of compounds in biological samples, and it is difficult to reproduce and regenerate the electrodes.

## 3. Conclusions

Despite the excellent sensitivity of traditional pathogen detection tools, using them to discover pathogens can be like looking for a needle in a haystack. The new generation of biosensors may be used to detect infections in various situations without the requirement for sample preparation. It is now possible to identify various illnesses using biosensors that may be applied to food, bodily fluids, and even objects’ surfaces. Despite having more sensitivity than electrochemical approaches, optical techniques are costly and challenging for most end users. Electrochemical methods, on the other hand, are more user-friendly, but their ability to detect diseases is still far from sufficient. Eventually, using a small biosensor near a patient’s bed, in a doctor’s office, or even at home may become viable with the commercialization of biosensor technology and the expansion of biosensor technology applications. 

## Figures and Tables

**Table 1:** Pathogen-based biorecognition elements.

Recognition Element	Advantages	Disadvantages	Reference(s)
Antibodies	High affinity High selectivity	Selectivity may be affected by antibody labeling Alteration of binding affinity to antigen Low temperature High cost	[47,48,49]
Carbohydrate binding proteins	Binding ligands unique to the Target organism Low cost High-yield automated synthesis	Pathogens detection abilities using electrochemical biosensors lack sufficient data	[41,50]
Oligosaccharides	Pathogens have receptors for Carbohydrate-specific trisaccharides Utilized with electrochemical biosensors	Limited selectivity Low affinity Carbohydrate–protein interaction	[41,51]
Oligonucleotides	Utilized with electrochemical biosensors Strong binding affinity and selectivity Low cost Feasible to extract and amplify particular binding sequences	Possibility of cross-reactions Lack of repeatability when using different procedures Degradation of aptamers	[52,53]
Cell-and molecular-imprinted polymers	Use morphology particular to target	Poor selectivity	[54,55]
Phages	Utilized with electrochemical biosensors Effective biorecognition component in water monitoring	High cost	[56,57]

**Table 2:** Chronological table of biosensor usage for bacterial or viral detection.

Bacteria/Virus	Method and Materials	Biorecognition Element	* LOD/LOQ	Year	Reference(s)
*E. coli*	Electrochemical impedance spectroscopy	Polyclonal anti-*E. coli*	10^4^ CFU/mL	2005	[58]
*V. cholerae*	Carbon electrode	Polyclonal anti-*V. cholerae*	8 CFU/mL	2006	[59]
*L. monocytogenes*	Electrode nanostructuring	Monoclonal anti-*L. monocytogenes*	4.7 × 10^2^ CFU/mL	2008	[60]
*S. typhimurium*	Ceramic electrodes	Anti-*S. typhimurium*	10^3^ CFU/mL	2009	[61]
West Nile virus (WNV)	Anodic stripping voltammetry	Monoclonal anti-WNV	0.02 viruses/mL	2009	[62]
*B. anthracis*	Ag electrode (Conductometry)	Monoclonal and polyclonal anti-*B. anthracis*	420 spores/mL	2009	[63]
*Campylobacter jejuni*	Nanoparticles on carbon electrode	Monoclonal anti-Flagellin A	10^3^ CFU/mL	2010	[64]
Bovine viral diarrhea virus (BVDV)	Nanofiber array electrode (Conductometry)	Monoclonal and polyclonal anti-BVDV	103 CCID **/mL	2010	[65]
*Helicobacter pylori*	Graphene interdigitated microelectrode array (Conductometry)	Odoranin-HP peptide	100 cells	2012	[66]
*L. innocua*	Phage	*L. innocua*-specific bacteriophage	1.1 × 10^4^–10^5^ CFU/mL	2012	[67]
*E. coli*	Cell- and molecularly imprinted polymers	Anti-*E. coli*	1.6 × 10^8^ Cells/mL	2014	[68]
*E. coli*	Composite on carbon electrode	Anti-*E. coli*	13 CFU/mL	2014	[69]
*S. typhimurium*	Electrochemical Impedance Spectroscopy (EIS)	Monoclonal anti*-S. typhimurium*	3 × 10^3^ CFU/mL	2015	[70]
*Enterococcus faecalis*	Carbon-based electrodes on Au electrode	Clavanin A peptide	10^3^ CFU/mL	2015	[71]
Dengue virus	AuNPs on Au electrode	Anti-DENV	---------------	2015	[72]
Norovirus	Au microelectrode (square wave voltammetry)	Anti-norovirus aptamer	10 PFU ***/mL	2016	[73]
Rotavirus	Electrochemical Impedance Spectroscopy (EIS) and nano structuring	Anti-rotavirus	2.3 PFU/mL R^2^ ****: 0.993	2016	[74]
*S. epidermidis*	Au microelectrode (Electrochemical Impedance Spectroscopy)	*S. epidermidis*-imprinted polymer film	10^3^ CFU/mL	2017	[75]
Influenza A virus (H1N1)	Oligosaccharides (PEDOT:PSS)	Hemagglutinin-specific trisaccharide ligand	0.13 HAU *****	2017	[51]
*E. coli* and human influenza A virus	Polymer electrode	Hemagglutinin-specific trisaccharide ligand	0.025 HAU	2018	[76]
*E. coli*	Carbohydrate binding proteins	Anti-*E. coli*	12 CFU/ml ----------------- 6.0 × 10^3^–9.2 × 10^7^ CFU/mL	2011 ------ 2019	[41,77]
SARS-CoV-2	CRISPER-Cas	---------------	Fold change: 10	2020	[78]
*S. typhimurium*	DNA functionalized	Amine labeled *S.* Typhi	6.8 × 10^−25^ molL^−1^	2022	[79]
SARS-CoV-2	Electrochemical immunosensor	SARS-CoV-2 spike protein	12 ng/mL–40 ng/mL	2022	[80]

* Limit of Detection (LOD), Limit of Quantification (LOQ). ** Cell Culture Infective Dose (CCID). *** Plaque-Forming Units (PFUs), Colony-Forming Units (CFUs). **** Linear Relationship: R^2^. ***** Haemagglutinin Unit: HAU.

## Data Availability

The data presented in this study are available within this article. Further inquiries may be directed to the authors.
